# Central venous minus arterial carbon dioxide pressure to arterial minus central venous oxygen content ratio as an indicator of tissue oxygenation: a narrative review

**DOI:** 10.5935/0103-507X.20200017

**Published:** 2020

**Authors:** Arnaldo Dubin, Mario Omar Pozo, Javier Hurtado

**Affiliations:** 1 Cátedra de Farmacología Aplicada, Facultad de Ciencias Médicas, Universidad Nacional de La Plata - La Plata, Argentina.; 2 Servicio de Terapia Intensiva, Sanatorio Otamendi - Buenos Aires, Argentina.; 3 Servicio de Terapia Intensiva, Hospital Británico - Buenos Aires, Argentina.; 4 Servicio de Terapia Intensiva, Hospital Español-ASSE - Montevidéu, Uruguai.; 5 Departamento de Fisiopatología, Facultad de Medicina, Universidad de la República - Montevidéu, Uruguai.

**Keywords:** Anaerobiosis, Respiration, Oxygenation, Carbon dioxide, Respiratory quotient, Critical illness, Anaerobiose, Respiração, Oxigenação, Dióxido de carbono, Quociente respiratório, Estado terminal

## Abstract

The central venous minus arterial carbon dioxide pressure to arterial minus central venous oxygen content ratio (P_cv-a_CO_2_/C_a-cv_O_2_) has been proposed as a surrogate for respiratory quotient and an indicator of tissue oxygenation. Some small observational studies have found that a P_cv-a_CO_2_/C_a-cv_O_2_ > 1.4 was associated with hyperlactatemia, oxygen supply dependency, and increased mortality. Moreover, P_cv-a_CO_2_/C_a-cv_O_2_ has been incorporated into algorithms for tissue oxygenation evaluation and resuscitation. However, the evidence for these recommendations is quite limited and of low quality. The goal of this narrative review was to analyze the methodological bases, the pathophysiologic foundations, and the experimental and clinical evidence supporting the use of P_cv-a_CO_2_/C_a-cv_O_2_ as a surrogate for respiratory quotient. Physiologically, the increase in respiratory quotient secondary to critical reductions in oxygen transport is a life-threatening and dramatic event. Nevertheless, this event is easily noticeable and probably does not require further monitoring. Since the beginning of anaerobic metabolism is indicated by the sudden increase in respiratory quotient and the normal range of respiratory quotient is wide, the use of a defined cutoff of 1.4 for P_cv-a_CO_2_/C_a-cv_O_2_ is meaningless. Experimental studies have shown that P_cv-a_CO_2_/C_a-cv_O_2_ is more dependent on factors that modify the dissociation of carbon dioxide from hemoglobin than on respiratory quotient and that respiratory quotient and P_cv-a_CO_2_/C_a-cv_O_2_ may have distinct behaviors. Studies performed in critically ill patients have shown controversial results regarding the ability of P_cv-a_CO_2_/C_a-cv_O_2_ to predict outcome, hyperlactatemia, microvascular abnormalities, and oxygen supply dependency. A randomized controlled trial also showed that P_cv-a_CO_2_/C_a-cv_O_2_ is useless as a goal of resuscitation. P_cv-a_CO_2_/C_a-cv_O_2_ should be carefully interpreted in critically ill patients.

## Abstract (pt)

A proporção entre pressão venosa central menos arterial de dióxido de carbono e conteúdo de oxigênio arterial menos venoso central (P_cv-a_CO_2_/C_a-cv_O_2_) foi proposta como marcador substituto para quociente respiratório e indicador de oxigenação tissular. Alguns pequenos estudos observacionais identificaram que P_cv-a_CO_2_/C_a-cv_O_2_ acima de 1,4 se associa com hiperlactatemia, dependência de suprimento de oxigênio e maior mortalidade. Mais ainda, a P_cv-a_CO_2_/C_a-cv_O_2_ foi incorporada a algoritmos para avaliação da oxigenação tissular e ressuscitação. Contudo, a evidência para estas recomendações é bastante limitada e de baixa qualidade. O objetivo desta revisão narrativa foi analisar as bases metodológicas, os fundamentos fisiopatológicos e a evidência experimental e clínica para dar suporte à utilização da P_cv-a_CO_2_/C_a-cv_O_2_ como marcador substituto para quociente respiratório. De um ponto de vista fisiopatológico, o aumento do quociente respiratório secundariamente a reduções críticas no transporte de oxigênio é um evento dramático e com risco à vida. Entretanto, este evento é facilmente observável e provavelmente não demandaria maiores monitoramentos. Visto que o início do metabolismo anaeróbico é indicado pelo aumento súbito do quociente respiratório e que a faixa normal do quociente respiratório é ampla, o uso do ponto de corte definido como 1,4 para P_cv-a_CO_2_/C_a-cv_O_2_ não faz sentido. Estudos experimentais demonstraram que a P_cv-a_CO_2_/C_a-cv_O_2_ é mais dependente de fatores que modificam a dissociação do dióxido de carbono da hemoglobina do que do quociente respiratório, e o quociente respiratório e P_cv-a_CO_2_/C_a-cv_O_2_ podem ter comportamentos distintos. Estudos conduzidos em pacientes críticos demonstraram resultados controvertidos com relação à capacidade da P_cv-a_CO_2_/C_a-cv_O_2_ para predizer o desfecho, hiperlactatemia, anomalias microvasculares e dependência de suprimento de oxigênio. Um estudo randomizado controlado também demonstrou que a P_cv-a_CO_2_/C_a-cv_O_2_ é inútil como alvo para ressuscitação. A P_cv-a_CO_2_/C_a-cv_O_2_ deve ser interpretada com cautela em pacientes críticos.

## INTRODUCTION

In critically ill patients, tissue hypoxia is a leading mechanism of multiple organ failure and death. Therefore, the detection and correction of anaerobic metabolism are crucial tasks. Unfortunately, gold standards for the assessment of tissue oxygenation are lacking. Some variables, which are commonly used in the monitoring of critically ill patients, might be misleading indicators of anaerobic metabolism. Lactate is an excellent predictor of outcome,^(1,2)^ but it is an unreliable marker of tissue hypoxia.^(3)^ Clinical monitoring of peripheral perfusion is an attractive and feasible approach but also has limitations.^(4)^ Although central venous oxygen saturation (S_cv_O_2_) and central venous minus arterial partial pressures of carbon dioxide (PCO_2_) difference (P_cv-a_CO_2_) can track changes in cardiac output, their role in the evaluation of the adequacy of tissue oxygenation is controversial.^(5,6)^ Regrettably, the evaluation of tissue PCO_2_ is no longer feasible, and the monitoring of microcirculation is still restricted to research.^(7)^ Hence, the search for new approaches is warranted.

**Figure 1:**
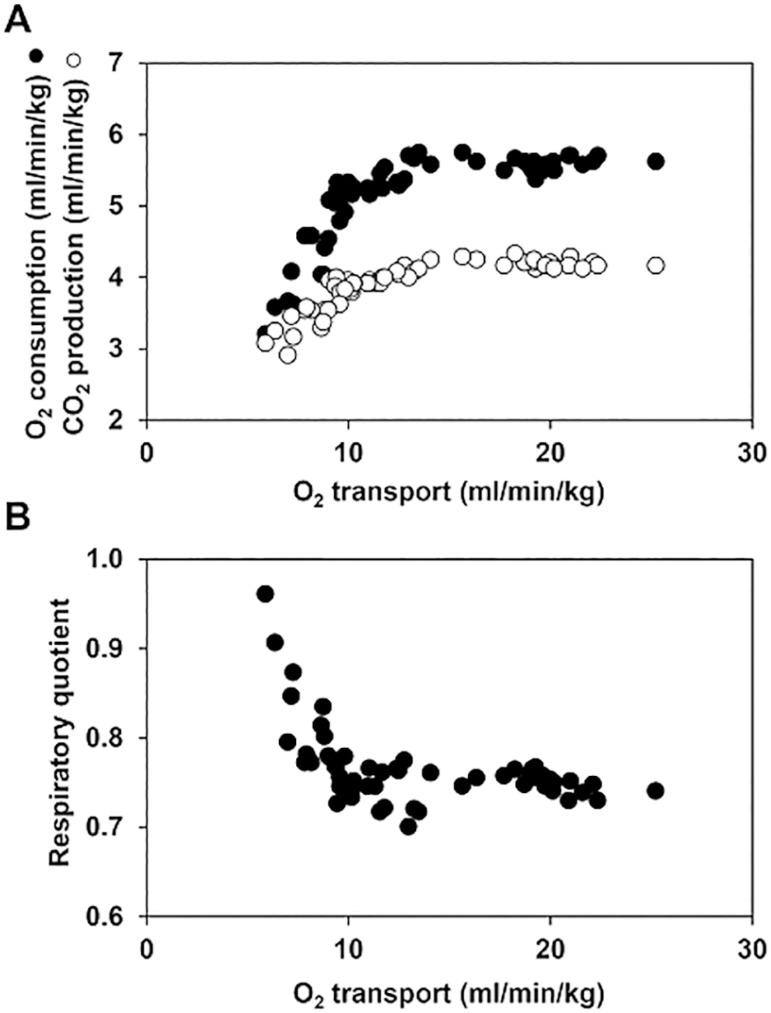
Oxygen consumption, carbon dioxide production, and respiratory quotient as function of oxygen transport. (A) Relationship of oxygen consumption and carbon dioxide production with oxygen transport. (B) Relationship of respiratory quotient with oxygen transport. Critical reductions in oxygen transport are associated with progressive decreases in oxygen consumption and carbon dioxide production. The reductions in carbon dioxide production are lower than those in oxygen consumption because of the beginning of anaerobic carbon dioxide production secondary to bicarbonate buffering of anaerobically generated protons. Consequently, sharp increases in respiratory quotient ensue. O_2_ - oxygen; CO_2_ - carbon dioxide.

In the physiology of exercise, the analysis of expired gases allows the identification of anaerobic metabolism. Increasing workloads are associated with parallel increases in carbon dioxide (CO_2_) production (VCO_2_) and O_2_ consumption (VO_2_). The slope of this relationship is the respiratory quotient (RQ = VCO_2_/VO_2_). The RQ remains initially constant under aerobic conditions. At some point, however, the increases in VCO_2_ exceed those in VO_2_, and the RQ increases. This inflection point corresponds with the development of hyperlactatemia and is known as the anaerobic threshold.^(8)^ In the context of the other physiological extreme, during oxygen supply dependency, the reductions in VO_2_ are higher than those in VCO_2_. Consequently, sharp elevations in RQ ensue^(9-11)^ (Figure 1). In both situations, anaerobic exercise and critical decreases in oxygen transport, the underlying phenomenon is the appearance of anaerobic VCO_2_ secondary to bicarbonate buffering of anaerobically generated protons. Thus, the increase in RQ indicates ongoing anaerobic metabolism.

Consequently, the measurement of RQ arises as an appealing approach for the identification of global tissue hypoxia. Nevertheless, metabolic carts are not usually available in the setting of the intensive care unit. In addition, the use of a high inspired oxygen fraction can interfere with the measurements.^(12)^ To solve this problem, some researchers have proposed a simplification of Fick’s equation adapted to CO_2_, the P_cv-a_CO_2_ to arterial minus central venous oxygen content ratio (P_cv-a_CO_2_/C_a-cv_O_2_), as a substitute for RQ. Some small observational studies have found that a P_cv-a_CO_2_/C_a-cv_O_2_ higher than 1.4 was associated with hyperlactatemia,^(13)^ oxygen supply dependency,^(14,15)^ and worse outcome.^(13)^ Moreover, P_cv-a_CO_2_/C_a-cv_O_2_ has been incorporated in algorithms for tissue oxygenation evaluation and resuscitation.^(16-18)^ However, the evidence for these recommendations is quite limited and of low quality.

The goal of this narrative review was to analyze the methodological bases, the pathophysiologic foundations, and the experimental and clinical evidence supporting the use of P_cv-a_CO_2_/C_a-cv_O_2_ as a surrogate for RQ. We comprehensively assessed the existing evidence for the association between P_cv-a_CO_2_/C_a-cv_O_2_ and outcomes in critically ill patients. We aimed to determine whether, in critically ill patients, increased P_cv-a_CO_2_/C_a-cv_O_2_ is associated with higher mortality and is a better predictor of outcome than arterial lactate. We also reviewed the role of P_cv-a_CO_2_/C_a-cv_O_2_ as a predictor of oxygen supply dependency and its use as a goal of resuscitation.

## METHODOLOGICAL CONCERNS

The use of P_cv-a_CO_2_/C_a-cv_O_2_ as a surrogate for RQ and tissue oxygenation relies on some assumptions. First, RQ is the ratio between VCO_2_ and VO_2_:

RQ = VCO_2_/VO_2_ (Equation 1)

(Equation 1) $$\mathrm{RQ}={\mathrm{VCO}}_{2}/{\mathrm{VO}}_{2}$$

According to Fick’s equation, this calculation can be rearranged as:

RQ = Q x C_mv-a_CO_2_/Q x C_a-mv_O_2_ (Equation 2)

(Equation 2) $$\mathrm{RQ}=Q\;x\;C_{\mathrm{mv}-a}{\mathrm{CO}}_{2}/Q\;x\;C_{a-\mathrm{mv}}O_{2}$$

where Q = cardiac output, C_mv-a_CO_2_ = mixed venous - arterial CO_2_ content difference, and C_a-mv_O_2_ = arterial - mixed venous oxygen content difference.

Next, an equivalence between mixed and central samples is assumed:

RQ = Q x C_cv-a_CO_2_/Q x C_a-cv_O_2_ (Equation 3)

(Equation 3) $$\mathrm{RQ}=Q\;x\;C_{\mathrm{cv}-a}{\mathrm{CO}}_{2}/Q\;x\;C_{a-\mathrm{cv}}O_{2}$$

where C_cv-a_CO_2_ = central venous - arterial CO_2_ content difference, and C_a-cv_O_2_ = arterial - central venous O_2_ content difference.

Then, the common factor (Q) is simplified in the numerator and denominator:

RQ = C_cv-a_CO_2_/C_a-cv_O_2_ (Equation 4)

(Equation 4) $$\mathrm{RQ}=C_{\mathrm{cv}-a}{\mathrm{CO}}_{2}/C_{a-\mathrm{cv}}O_{2}$$

Finally, since the calculation of CO_2_ content is not straightforward, CO_2_ content is replaced by CO_2_ pressure. This assumption is based on the fact that CO_2_ content and pressure are linearly correlated over the physiological range of CO_2_ content:

RQ = P_cv-a_CO_2_/C_a-cv_O_2_ (Equation 5)

(Equation 5) $$\mathrm{RQ}=P_{\mathrm{cv}-a}{\mathrm{CO}}_{2}/C_{a-\mathrm{cv}}O_{2}$$

Unfortunately, some of these assumptions are problematic, such as the use of CO_2_ pressure instead of content^(19,20)^ and the lack of interchangeability between central and mixed venous samples.^(21)^ In addition, the use of a defined cutoff of P_cv-a_CO_2_/C_a-cv_O_2_ for the identification of tissue hypoxia is also questionable.^(9-11)^ In the following paragraphs, we reviewed these and other methodological issues.

### The use of carbon dioxide pressure instead of content in the calculation of the ratio

**Figure 2:**
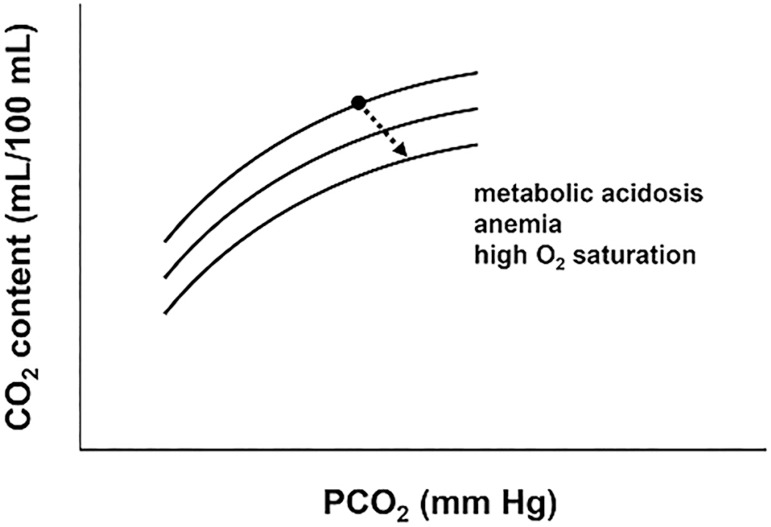
Relationship between carbon dioxide content and pressure (carbon dioxide dissociation curve). For the same partial pressure of carbon dioxide, anemia, metabolic acidosis, and elevated oxygen saturation (Haldane effect) are associated with decreased carbon dioxide content. CO_2_ - carbon dioxide; O_2_ - oxygen; PCO_2_ - partial pressure of carbon dioxide.

The relationship between CO_2_ content and pressure is complex. In general terms, the relationship is curvilinear. This implies that for the highest range of CO_2_ content, the relationship becomes flatter. In this way, further increases in CO_2_ content induce a larger increase in PCO_2_. The CO_2_ dissociation curve can be modified by changes in base excess, hemoglobin levels, and oxygen saturation (Haldane effect) (Figure 2). These factors can significantly modify P_cv-a_CO_2_/C_a-cv_O_2_, even in the absence of alterations in RQ and tissue oxygenation.^(19)^

Theoretically, these drawbacks of P_cv-a_CO_2_/C_a-cv_O_2_ might be overcome by the use of equation 3. Nevertheless, any algorithm for the calculation of CO_2_ content can be misleading. For example, the most commonly used method results in 95% limits of agreement between calculated and manometrically measured CO_2_ content of 4.66mL/100mL.^(22)^ This value is unacceptably high, especially taking into account the error propagation related to the additional calculation of venous - arterial CO_2_ content difference. Consequently, the method frequently produces unreliable negative values of C_mv-a_CO_2_.^(23)^

**Figure 3:**
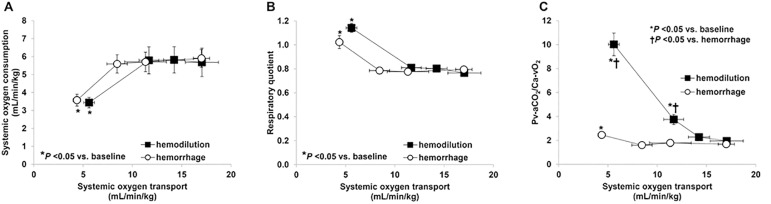
Oxygen consumption, respiratory quotient, and venoarterial partial pressure of carbon dioxide difference with arteriovenous oxygen content difference ratio as functions of oxygen transport . Relationship of oxygen transport with oxygen consumption (A), respiratory quotient (B), and venoarterial partial pressure of carbon dioxide difference with arteriovenous oxygen content difference ratio (P_v-a_CO_2_/C_a-v_O_2_). Oxygen consumption decreased and respiratory quotient increased only in the last step of hemodilution and hemorrhage. In the context of hemodilution, the increase in P_v-a_CO_2_/C_a-v_O_2_ was higher than in the hemorrhage condition and appeared before the development of oxygen supply dependency.

Source: reproduced with permission of Dubin et al.^(19)^

**Figure 4:**
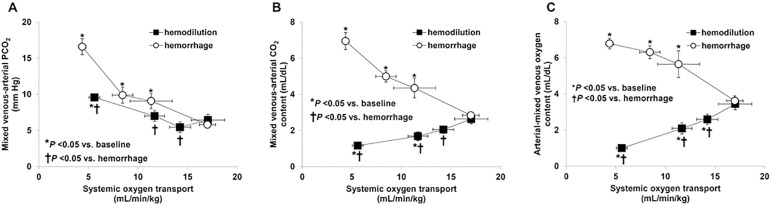
Oxygen consumption, respiratory quotient, and venoarterial partial pressure of carbon dioxide difference with arteriovenous oxygen content difference ratio as functions of oxygen transport. Relationship of oxygen transport with venoarterial partial pressure of carbon dioxide difference (P_v-a_CO_2_) (A), venoarterial carbon dioxide content difference (C_v-a_CO_2_) (B), and arteriovenous oxygen content difference (C_a-v_O_2_). Hemodilution produced opposite effects on Pv-aCO2 and Cv-aCO2. C_v-a_CO_2_ decreased in the context of hemodilution and increased in the hemorrhage condition. These changes are the underlying explanation for the different behavior of P_v-a_CO_2_/C_a-v_O_2_ in both groups. CO_2_ - carbon dioxide. Source: reproduced with permission of Dubin et al.^(19)^

An experimental study addressed the methodological pitfalls associated with P_mv-a_CO_2_/C_a-mv_O_2_ as a surrogate for RQ.^(19)^ In this study, P_mv-a_CO_2_/C_a-mv_O_2_, RQ, and their determinants were measured during stepwise reductions of oxygen transport (DO_2_) induced by hemorrhage or hemodilution. The correlation between P_mv-a_CO_2_/C_a-mv_O_2_ and RQ was significant but poor. Moreover, in the context of hemodilution, P_mv-a_CO_2_/C_a-mv_O_2_ increased even before the decrease in VO_2_ and the increase in RQ (Figure 3). This finding was related to the opposite effects of hemoglobin reduction on P_mv-a_CO_2_ and C_a-mv_O_2_. P_mv-a_CO_2_ increased as a consequence of the effects of anemia on the CO_2_ dissociation curve, while C_a-mv_O_2_ decreased as a result of the increase in the oxygen extraction ratio (Figure 4). In addition, in the last step of DO_2_ reduction and despite similar degrees of tissue hypoxia and elevations in RQ, P_mv-a_CO_2_/C_a-mv_O_2_ disproportionally increased in the context of hemodilution compared to the hemorrhage condition because of the aforementioned factors. Moreover, a multiple linear regression model identified hemoglobin, metabolic acidosis, the Haldane effect, the position in the flattened portion of the CO_2_ dissociation curve, and RQ as independent determinants of P_mv-a_CO_2_/C_a-mv_O_2_. Although P_mv-a_CO_2_/C_a-mv_O_2_ was found to be dependent on RQ, this was its weakest determinant.

Given that metabolic acidosis is a key determinant of P_mv-a_CO_2_/C_a-mv_O_2_, the relationship between this variable and lactate is complicated. Both variables can be increased as an expression of anaerobic metabolism. On the other hand, hyperlactatemia that results from the activation of aerobic glycolysis can increase P_mv-a_CO_2_/C_a-mv_O_2_, even in the absence of tissue hypoxia. For example, after blood retransfusion in experimental hemorrhagic shock, VO_2_ and RQ normalize, but P_mv-a_CO_2_/C_a-mv_O_2_ remain high as a probable consequence of persistent hyperlactatemia.^(20)^ Although P_mv-a_CO_2_/C_a-mv_O_2_ has been suggested as a tool to assert the source of lactate,^(18)^ the results of this experimental study suggest that this approach might be misleading.

### The poor agreement between central and mixed venous samples

Another methodological concern is the lack of interchangeability between central and mixed venous samples for the different calculations. The issue of the agreement between mixed venous and central O_2_ saturation has been extensively addressed. Although the agreement is poor, a small study advocated that both variables have similar behavior.^(24)^ In contrast, a multicenter study comprehensively showed that both variables not only have poor agreement but also might differ in the direction of their changes.^(25)^ Furthermore, a recent study showed that the problem is even worse for CO_2_-derived variables: 95% limits of agreement for venous-arterial PCO_2_ and its ratio to arterial-venous O_2_ content were 8mmHg and 1.48, respectively, which are unacceptably high.^(21)^

### The use of a particular cutoff of P_cv-a_ CO_2_/C_a-cv_ O_2_ for the diagnosis of anaerobic metabolism

In addition, the identification of anaerobic metabolism based on a cutoff value of P_cv-a_CO_2_/C_a-cv_O_2_ is arguable. Since the beginning of anaerobic metabolism is indicated by sharp increases in RQ, not by a particular threshold,^(9-11)^ similar criteria should be considered for P_cv-a_CO_2_/C_a-cv_O_2_. This is especially relevant taking into account the fact that a normal RQ ranges from 0.67 to 1.30.^(26)^ The wide normal limits of RQ are mainly dependent on energetic oil. Diets based only on carbohydrates and overfeeding consistently increased RQ, while fat-based diets and fasting decreased RQ.

Accordingly, a clinical study showed that the ability of the absolute value of RQ to predict death or low cardiac output syndrome was lower than that of lactate in cardiac surgery patients.^(27)^ Although RQ was higher in nonsurvivors (0.83 ± 0.08 *versus* 0.75 ± 0.08; p = 0.02), values were in the normal range. Therefore, defined cutoffs of P_cv-a_CO_2_/C_a-cv_O_2_ or RQ should not be used for the detection of anaerobic metabolism; sudden increases in these parameters should be used instead.

### The use of calculated instead of measured oxygen saturation for P_cv-a_ CO_2_/C_a-cv_ O_2_

Another relevant limitation of some studies was that the computation of P_cv-a_CO_2_/C_a-cv_O_2_ was based on values of oxygen saturation calculated from blood gases and oxyhemoglobin dissociation curves instead of measurements performed by co-oximetry.^(23,28-30)^ The calculated O_2_ saturation does not agree with the measured values. In addition, the measurement error is propagated in the calculation of P_cv-a_CO_2_/C_a-cv_O_2_. Additionally, paired measurements of P_cv-a_CO_2_/C_a-cv_O_2_ in the same analyzer had poor reproducibility with 95% limits of agreement of 1.22.^(31)^

### The physiological feasibility of increased P_cv-a_ CO_2_/C_a-cv_ O_2_ as a reflection of tissue hypoxia in critically ill patients

In experimental models, the increase in RQ is a dramatic event associated with impending death. For example, during progressive hemodilution, RQ increases only when hemoglobin reaches 1.2g%. Likewise, during progressive bleeding, RQ increases when the mean arterial pressure is lower than 30mmHg.^(11)^ These are extreme and obvious conditions that do not require additional monitoring or sensitive assessment of tissue oxygenation to be identified. Accordingly, high values of P_cv-a_CO_2_/C_a-cv_O_2_ in patients with otherwise stable conditions might seldom reflect global anaerobic metabolism but rather the presence of factors that modify the dissociation of CO_2_ from hemoglobin. This has been shown in experimental models, in which RQ and P_mv-a_CO_2_/C_a-mv_O_2_ are poorly correlated. In these cases, the presence of anemia, metabolic acidosis, or the Haldane effect explained the findings of increased P_mv-a_CO_2_/C_a-mv_O_2_, even when VO_2_ and RQ were normal.^(19)^ In addition, at higher levels of CO_2_ content, the linear relationship between CO_2_ content and pressure is progressively lost, and minor changes in CO_2_ content may be associated with larger changes in PCO_2_. In patients, a direct comparison between P_mv-a_CO_2_/C_a-mv_O_2_ or P_cv-a_CO_2_/C_a-cv_O_2_ and RQ has not yet been performed. Therefore, values of P_cv-a_CO_2_/C_a-cv_O_2_ should be interpreted cautiously in clinically stable patients.

### The clinical usefulness of P_cv-a_ CO_2_/C_a-cv_ O_2_

Although P_cv-a_CO_2_/C_a-cv_O_2_ might fail to reflect the actual value of RQ, it might still reflect the severity of the critical illness because it is a compound variable, which is partially dependent on hemoglobin and base excess. Consequently, anemia and metabolic acidosis can produce high P_cv-a_CO_2_/C_a-cv_O_2_ by themselves and can be indicators of a severe condition or predictors of worse outcome. Thus, the presence of anemia and metabolic acidosis might be responsible for the predictive ability of P_cv-a_CO_2_/C_a-cv_O_2_. Next, we discuss the controversies about P_cv-a_CO_2_/C_a-cv_O_2_ as a predictor of outcome, oxygen supply dependency and microcirculatory alterations and as a goal of resuscitation.

### P_cv-a_ CO_2_/C_a-cv_ O_2_ as a predictor of outcome and hyperlactatemia

Several years ago, a retrospective study of 89 patients monitored with a pulmonary catheter found that P_mv-a_CO_2_/C_a-mv_O_2_ was a sensitive predictor of hyperlactatemia, with an area under receiving operating characteristic (AUROC) curve of 0.85.^(13)^ P_mv-a_CO_2_/C_a-mv_O_2_ was also higher in hyperlactatemic patients than in normolactatemic patients (2.0 ± 0.9 *versus* 1.1 ± 0.6). Additionally, 30-day survival was higher for patients with P_mv-a_CO_2_/C_a-mv_O_2_ < 1.4 than for patients with a ratio ≥ 1.4 (38 *versus* 20%). Despite these findings, P_mv-a_CO_2_/C_a-mv_O_2_ was not significantly different between nonsurvivors and survivors (1.7 ± 1.0 *versus* 1.3 ± 0.5). On the other hand, lactate was lower in survivors (2.0 ± 1.5 *versus* 5.4 ± 6.1mmol/L). Although lactate and P_mv-a_CO_2_/C_a-mv_O_2_ were correlated and P_mv-a_CO_2_/C_a-mv_O_2_ showed some association with outcome, lactate was a better predictor of outcome in this study.^(13)^ Similarly, in 135 patients with septic shock, P_mv-a_CO_2_/C_a-mv_O_2_ and lactate had a distinct time course in survivors and nonsurvivors, but only C_mv-a_CO_2_/C_a-mv_O_2_ and lactate, but not P_mv-a_CO_2_/C_a-mv_O_2_, were related to the outcome.^(28)^

In another study performed with 50 patients with shock, P_cv-a_CO_2_/C_a-cv_O_2_ and lactate at the beginning of the study were lower in survivors than in nonsurvivors. Lactate, however, showed a better prognostic ability (AUROC curve of 0.73 and 0.81).^(29)^ A retrospective series of 144 patients with septic shock found that P_cv-a_CO_2_/C_a-cv_O_2_ and lactate showed a similar ability to predict mortality and organ failure, but their combination was superior.^(30)^ In another observational study in 35 patients with septic shock, P_cv-a_CO_2_/C_a-cv_O_2_ was a strong predictor of lactate behavior, and both variables were associated with outcome.^(32)^

In contrast, other studies have shown that P_cv-a_CO_2_/C_a-cv_O_2_ has a poor association with either lactate or mortality. In a prospective multicenter cohort study that recruited 363 patients with septic shock, P_cv-a_CO_2_/C_a-cv_O_2_ could not distinguish patients with high lactate levels or poor lactate clearance from patients with low lactate levels or proper lactate clearance.^(33)^ In another observational study of 23 septic patients, neither P_cv-a_CO_2_/C_a-cv_O_2_ nor P_mv-a_CO_2_/C_a-mv_O_2_ distinguished survivors from nonsurvivors.^(21)^

In brief, there are conflicting results about the relationship between P_cv-a_CO_2_/C_a-cv_O_2_ and mortality. It appears that a high P_cv-a_CO_2_/C_a-cv_O_2_ has some prognostic implications that seem similar to those of lactate. There are also conflicting reports about the relationship between P_cv-a_CO_2_/C_a-cv_O_2_ and lactate. Nevertheless, there is great heterogeneity among the studies.

### P_cv-a_ CO_2_/C_a-cv_ O_2_ as a predictor of oxygen supply dependency

The increase in VO_2_ in response to elevated DO_2_ is characterized as oxygen supply dependency. Although the oxygen supply dependency might reveal the presence of an oxygen debt, its actual meaning is controversial. Since both VO_2_ and DO_2_ are usually calculated from a shared variable (cardiac output) and the magnitude of change of the calculated variables is also small, there is a considerable risk of mathematical coupling of data. Therefore, oxygen supply dependency is sometimes not a real fact but an artifact. Nevertheless, different studies have aimed to show that P_cv-a_CO_2_/C_a-cv_O_2_ is a predictor of oxygen supply dependency.

In 25 patients with shock, in whom cardiac output was increased in response to 500mL of saline solution, VO_2_ increased in 14 patients and remained unchanged in 11 patients.^(14)^ Lactate (5.5 ± 4.0 *versus* 2.3 ± 1.1mmol/L) and P_cv-a_CO_2_/C_a-cv_O_2_ (2.3 ± 0.8 *versus* 1.3 ± 0.5) were higher in patients with oxygen supply dependency. Both variables, lactate and P_cv-a_CO_2_/C_a-cv_O_2_, showed high areas under the receiving operating characteristic (AUROC) curves to predict the increase in VO_2_ (0.94 ± 0.05 and 0.91 ± 0.06, respectively). Another study, performed with 51 fluid-responsive patients with septic shock, also showed increased levels of lactate and P_cv-a_CO_2_/C_a-cv_O_2_ in patients with oxygen supply dependency, but lactate had a lower AUROC (0.745 *versus* 0.965).^(15)^ In contrast, in 92 fluid responders admitted to a cardiothoracic ICU, P_cv-a_CO_2_/C_a-cv_O_2_ failed to predict the increase in VO_2_ (AUROC = 0.52).^(34)^ In another small study, in 17 cardiac surgery patients, P_cv-a_CO_2_/C_a-cv_O_2_ was also unable to predict oxygen supply dependency (AUROC = 0.64).^(35)^

Therefore, the results are inconclusive. Furthermore, in these studies, VO_2_ was calculated from central instead of mixed venous samples, which generates further methodological uncertainties about the interpretation of the results.

### P_cv-a_ CO_2_/C_a-cv_ O_2_ as a predictor of microcirculatory alterations

An observational study described a correlation between C_mv-a_CO_2_/C_a-mv_O_2_ and the sublingual proportion of perfused vessels in patients with septic shock.^(23)^ Another clinical study, however, did not find any correlation of P_cv-a_CO_2_/C_a-cv_O_2_ or P_mv-a_CO_2_/C_a-mv_O_2_ with sublingual microcirculation.^(21)^

### P_cv-a_ CO_2_/C_a-cv_ O_2_ as a goal of resuscitation

Only one study has assessed the usefulness of P_cv-a_CO_2_/C_a-cv_O_2_ as a goal of resuscitation.^(36)^ In a randomized controlled trial, 228 patients were assigned to P_cv-a_CO_2_/C_a-cv_O_2_- or S_cv_O_2_-targeted resuscitation. There were no differences in mortality, organ failure, length of stay, or other secondary outcomes.

## CONCLUSIONS

The clinical use of P_cv-a_CO_2_/C_a-cv_O_2_ as a surrogate for RQ is controversial. First, the increase in respiratory quotient secondary to critical reductions in oxygen transport is a life-threatening and dramatic but easily noticeable event that probably does not require further monitoring. Since the beginning of anaerobic metabolism is indicated by the sudden increase in respiratory quotient and the normal range of respiratory quotient is wide, the use of a defined cutoff of 1.4 for P_cv-a_CO_2_/C_a-cv_O_2_ is meaningless. P_cv-a_CO_2_/C_a-cv_O_2_ is more dependent on factors that modify the dissociation of carbon dioxide from hemoglobin than on respiratory quotient. Experimental studies have shown that RQ and P_cv-a_CO_2_/C_a-cv_O_2_ might display distinct behaviors in different models. Clinical studies in critically ill patients have shown controversial results regarding the ability of P_cv-a_CO_2_/C_a-cv_O_2_ to predict outcomes, hyperlactatemia, microvascular abnormalities, and oxygen supply dependency. A randomized controlled trial also showed that P_cv-a_CO_2_/C_a-cv_O_2_ is useless as a goal of resuscitation. Consequently, P_cv-a_CO_2_/C_a-cv_O_2_ should be carefully interpreted in critically ill patients.
